# Identifying community spread of COVID-19 via a free drive-through screening event

**DOI:** 10.1017/ice.2020.341

**Published:** 2020-07-20

**Authors:** Marci L. Drees, Mia A. Papas, Terri E. Corbo, Kimberly D. Williams, Sharon T. Kurfuerst

**Affiliations:** 1 Quality and Safety Administration, ChristianaCare, Wilmington, Delaware; 2 Value Institute, ChristianaCare, Wilmington, Delaware; 3 Clinical Essential Services Administration, ChristianaCare, Wilmington, Delaware; 4 Chief Operating Officer, ChristianaCare, Wilmington, Delaware

*To the Editor*—The lack of resources for and limitations placed upon testing for the 2019 novel coronavirus disease (COVID-19) have been well documented.^1–3^ On March 12, 2020, the governor of Delaware declared a state of emergency with identification of the first local cases of COVID-19, which led to an increase in testing interest among the public. To offload emergency department demand, on March 13, Delaware’s largest private not-for-profit healthcare system conducted a 1-day drive-through testing event.

The 4-hour event was held in a public space separate from the healthcare system’s campuses. Staff wore appropriate personal protective equipment (gowns, gloves, and masks with eye protection). They changed gloves and performed hand hygiene between each participant. Participants remained in their vehicles and were registered utilizing paper forms. Insurance information was not collected. All were provided instructions regarding self-isolation until results were available. At the time, testing was only available via the state public health laboratory and was restricted to those demonstrating symptoms and having a defined risk factor, such as travel or contact with a known case. For this event, asymptomatic persons could not be tested, but risk factors were not required to be present. All specimens were sent to a reference laboratory (Viracor Eurofins, Lee’s Summit, MO) and no other viral testing was performed. Negative cases were notified of their results by text, e-mail, or phone, according to their preference; positive cases were called, were provided with self-isolation and other instructions, and were reported to the Delaware Division of Public Health for follow-up.

Participants provided demographic information and attested to the following factors: (1) presence of symptoms: fever and/or chills, difficulty breathing, cough, or sore throat; (2) risk factors such as close contact with a known case or travel from an area with COVID-19 transmission during that period (eg, China, Italy, Iran, Japan, South Korea; Washington or New York); and/or (3) under self-quarantine or being monitored by a health department. Descriptive statistics examined the prevalence of demographic characteristics, clinical characteristics, and risk factors in this population, including χ^2^ statistics to assess bivariate associations. Analyses were conducted using Stata version 15.1 software (StataCorp, College Station, TX).

We screened 539 individuals; 2 results were invalid, leaving 537 individuals with completed testing. Table 1 reports the demographic and clinical characteristics of the positive cases and the total participant population. One-quarter of those tested reported only 1 symptom, while nearly half indicated having 2 symptoms. We detected no differences in symptoms or risk factor profiles by gender, age, or state of residence (all *P* > .05).

**Table 1.** Demographic and Clinical Characteristics of 537 Participants in COVID-19 Drive-through Testing Event, March 13, 2020

Characteristic	Positive Cases N = 12		Total Population N = 537	
	No.	%	No.	%
**Gender**				
Female	5	41	306	56.9
Male	7	58	231	43.0
**Age, y**				
0–9	0	0	17	3.1
10–19	1	8	29	5.4
20–29	1	8	83	15.4
30–39	2	16	123	22.9
40–49	2	16	91	16.9
50–59	3	25	92	17.1
60–69	3	25	72	13.4
70–79	0	0	26	4.8
≥80	0	0	4	0.7
**State of residence**				
Delaware	8	66	407	75.7
Other	4	33	130	24.2
**Clinical symptom present**				
Fevers/Chills	10	83	304	56.6
Cough	9	75	428	79.7
Difficulty breathing	2	16	174	32.4
Sore throat	3	25	236	43.9
1 symptom	3	25	128	23.8
2 symptoms	6	50	240	44.6
3 symptoms	3	25	126	23.4
All 4 symptoms	0	0	43	8.0
**Risk factor present**				
Traveled to area with local transmission	1	8	67	12.4
Close contact with a positive case	0	0	56	10.4
Quarantined or monitored by health department	1	8	55	10.2
No risk factors	10	83	379	70.5
Only 1 risk factor	2	16	139	25.8
2 risk factors	0	0	18	3.3
All 3 risk factors	0	0	1	0.19

Of the 537 persons tested, we confirmed 12 positive cases, for a COVID-19 prevalence rate of 2.2%. Fever and/or chills were the most commonly reported symptoms, followed by cough, sore throat, and difficulty breathing. Of the 12 positive cases, 2 reported a positive risk-factor profile. There were no statistically significant differences in demographic characteristics or risk-factor profiles between positive and negative cases. Positive cases were more likely to report fever and/or chills than were negative cases (83% vs 56%, respectively; *P* = .05).

This drive-through event documented community transmission for the first time in our state. In the 2 months after the event was held (as of May 28, 2020), 9,096 additional positive cases were identified in Delaware, with numbers increasing daily. Public screening events, including drive-through testing, are effective strategies for identifying disease transmission and providing additional details on the ever-changing risk of COVID-19 to the community.^4^

These events are also critical for identifying individuals at high risk for transmission, but they would not have been identified with the strict criteria used by the state and federal government at the time. Of the 12 positive cases identified, 1 was a teacher and 1 was a healthcare worker; neither qualified for testing by the state health department. Both individuals, if not identified, could have unknowingly infected many susceptible individuals. As our understanding of the disease deepens, it is critical to relax strict testing criteria to identify both symptomatic and asymptomatic individuals who can unknowingly transmit this virus.

Everyone tested at this event had at least 1 clinical symptom. Although fever and chills were more likely to be reported among cases than noncases, we detected no difference in the total number of symptoms reported between these 2 groups, with approximately one-quarter reporting 3 or more symptoms. Clearly, many other respiratory infections are cocirculating, although we did not conduct additional testing to confirm other pathogens. This highlights the ongoing challenge of differentiating COVID-19 from more common respiratory illnesses in the absence of widespread testing. As we enter the fall, officials estimate that we may see a second wave of COVID-19 occur simultaneously with other more common respiratory illnesses including influenza. Healthcare systems will need to prepare for the impact of this on hospital resources. Similar symptomology and a lack of testing will lead to challenges in differentiating COVID-19 from other respiratory illnesses.

This public event was free, financially supported by a private healthcare system, and set up in <24 hours. The need for such an event to document community spread of COVID-19 speaks to the failure in the United States to support a public health system that could provide easily available testing to identify, isolate, and halt community spread of this highly contagious disease. Both private and public health systems need to work together to expand capacity for testing, enabling the public health system to perform the necessary isolation and contact tracing that are required to slow and eventually stop the continued spread of COVID-19.
